# The rostral migratory stream generates hippocampal CA1 pyramidal-like neurons in a novel organotypic slice co-culture model

**DOI:** 10.1242/bio.012096

**Published:** 2015-09-04

**Authors:** Ilyas Singec, Rolf Knoth, Imre Vida, Michael Frotscher

**Affiliations:** 1 Institute of Anatomy and Cell Biology, Albert-Ludwigs-University Freiburg, D-79104 Freiburg, Germany; 2 Department of Neuropathology, Albert-Ludwigs-University Freiburg, D-79106 Freiburg, Germany; 3 Institute for Integrative Neuroanatomy, Charité, D-10117 Berlin, Germany; 4 Center for Molecular Neurobiology (ZMNH), University Medical Center Hamburg-Eppendorf, D-20251 Hamburg, Germany

**Keywords:** Cellular plasticity, Microenvironment, Neuronal differentiation, Organotypic slice culture, Pyramidal neuron, Rostral migratory stream

## Abstract

The mouse subventricular zone (SVZ) generates large numbers of neuroblasts, which migrate in a distinct pathway, the rostral migratory stream (RMS), and replace specific interneurons in the olfactory bulb (OB). Here, we introduce an organotypic slice culture model that directly connects the RMS to the hippocampus as a new destination. RMS neuroblasts widely populate the hippocampus and undergo cellular differentiation. We demonstrate that RMS cells give rise to various neuronal subtypes and, surprisingly, to CA1 pyramidal neurons. Pyramidal neurons are typically generated before birth and are lost in various neurological disorders. Hence, this unique slice culture model enables us to investigate their postnatal genesis under defined *in vitro* conditions from the RMS, an unanticipated source for hippocampal pyramidal neurons.

## INTRODUCTION

Neural stem cells (NSCs) of the SVZ reside in the lateral walls of the lateral ventricles and the dentate gyrus (DG) of the hippocampus and generate new neurons throughout rodent life (Lledo et al., 2008). Postnatal hippocampal neurogenesis produces granule cells in the DG, and their differentiation process has been characterized in rodents and humans (Kempermann et al., 2004; Knoth et al., 2010; Aimone et al., 2014; Jessberger and Gage, 2014). Immature granule cells migrate only a short distance in the DG until they reach their final position in the granule cell layer (GCL). In contrast, NSCs of the SVZ generate large numbers of neuroblasts that tangentially migrate over a long distance (several millimeters) to the OB and replace different types of interneurons (Luskin, 1993; Lois et al., 1996; Brill et al., 2009; Merkle et al., 2007, 2014). This unique pathway that connects the SVZ with the OB is called the RMS. Neuroblasts of the RMS are proliferative and display a specific mode of migration, which has been described as “homophilic chain migration” (Lois et al., 1996; Doetsch and Alvarez-Buylla, 1996; Wichterle et al., 1997). Proper migration of RMS cells is coordinated by a complex interplay of cellular and extracellular matrix components and secreted factors. Collectively, these chemo-attractant and chemo-repulsive cues maintain the highly dynamic structure of the SVZ-RMS system and ensure coordinated long-distance migration and cell turnover in the OB (Wu et al., 1999; Hack et al., 2002; Ng et al., 2005; Lledo et al., 2008; Rutishauser, 2008; Snapyan et al., 2009; Fuentealba et al., 2012; García-González et al., 2014; Girard et al., 2014).

Recruitment of endogenous neural stem/progenitor cells and transplantation of *ex vivo* generated neural cells hold great promise for regenerative medicine (Singec, 2013; Aimone et al., 2014). Traditional cell grafting experiments require enzyme-based single cell dissociation, which typically disrupts the physiological state of cell-cell and cell-matrix interactions and alters cell surface molecules and secreted factors. Hence, important environmental cues and molecular signals are irreversibly lost in ordinary neural transplantation studies. This shortcoming may compromise the analysis of the full developmental potential of grafted cells. Successful cell replacement strategies will require a better understanding of cell autonomous and non-cell autonomous mechanisms by which endogenous or grafted progenitors and their cellular progeny are regulated. Considering the complexity and importance of the tissue microenvironment for neuronal migration, survival, differentiation, and synaptic integration, we set out to establish a model system that maintains the molecular milieu of both donor and host tissues in an experimentally accessible *in vitro* assay. Specifically, to test the cellular plasticity of the RMS under organotypic conditions, we established a novel slice co-culture model by surgically removing the OB and connecting the RMS directly to the hippocampus as a new destination. We demonstrate that large numbers of RMS cells populate the different hippocampal regions and differentiate into various neuronal cell types. Unexpectedly, we found that RMS cells can integrate into the hippocampal CA1 region and properly differentiate into pyramidal neuron-like cells. These findings suggest the existence of molecular cues and environmental signals that can reprogram the cellular fate of olfactory neuroblasts into appropriate hippocampal principal neurons.

## RESULTS AND DISCUSSION

**Fig. 1.**
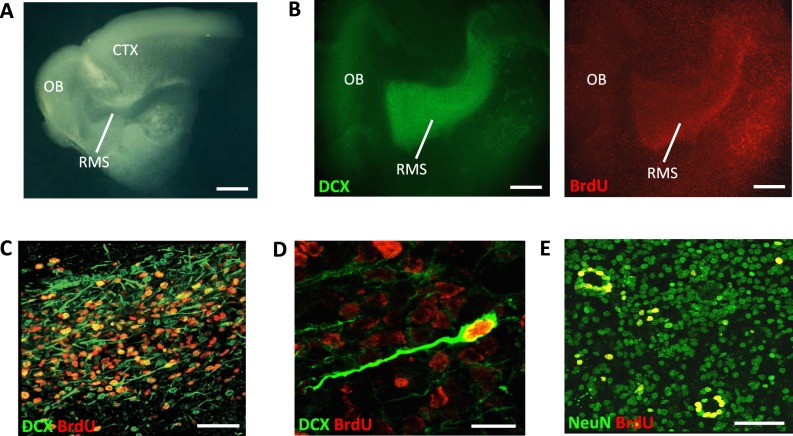
**Organotypic slice cultures of the RMS-OB pathway.** (A) Representative overview showing a forebrain slice cultured for 30 days. Note that the RMS and OB are well maintained. CTX, cortex; scale bar, 400 μm. (B) DCX and BrdU expression in the RMS. Scale bar, 200 μm. (C) Numerous DCX and BrdU labeled cells are present in the RMS. Scale bar, 50 μm. (D) Higher magnification of the RMS shows a BrdU-labeled migratory neuroblast with a leading process and DCX expression. Scale bar, 10 μm. (E) Analysis of the OB shows BrdU-labeled cells co-localizing the neuronal marker NeuN (yellow). Scale bar, 20 μm.

Organotypic slice cultures derived from different brain regions (e.g. hippocampus, cerebellum, spinal cord) have been used in previous studies and enable important experiments otherwise not feasible in the intact brain or primary cultures containing dissociated cells (Gähwiler et al., 1997; De Marchis et al., 2001; Tanvig et al., 2009). For instance, in an earlier study using organotypic slice co-cultures, we demonstrated that reelin acts as a positional signal for dentate granule cells (Zhao et al., 2004). In the present study, we isolated the forebrain from early postnatal mice and sectioned them sagittally into 350-μm-thick slices by using a tissue chopper. Only slices that included the *en bloc* RMS-OB system were selected and cultured on semiporous membranes for up to 5 weeks according to the interface method (Stoppini et al., 1991). During this culture period the RMS maintained its anatomical organization and was readily identifiable by its distinct lucent-like structure as compared to the surrounding tissues (Fig. 1A). Immunohistochemistry and confocal microscopic analysis of the cultured RMS showed the presence of large numbers of cells with typical morphologies of migratory neuroblasts such as prominent leading process and spindle-shaped cell bodies (Fig. 1B-D). These cells were immunopositive for doublecortin (DCX), a marker for immature migratory neuroblasts (Knoth et al., 2010). At 10 days *in vitro* (DIV), slice cultures were incubated for 24 h in 5-bromo-2′-deoxyuridine (BrdU), a well-established marker for dividing cells. These experiments demonstrated that a large fraction of migratory cells in the RMS were proliferative (Fig. 1B-D) resembling their *in vivo* behavior. In parallel experiments, BrdU-treated slices were kept in culture and fixed at DIV30 and the OB was analyzed using the neuronal marker NeuN. These experiments showed cellular co-localization of BrdU and NeuN in the different layers of the OB suggesting ongoing neurogenesis *in vitro* (Fig. 1E). Together, these results indicate that the RMS-OB pathway is functionally maintained in slice cultures and amenable for *in vitro* studies.

**Fig. 2.**
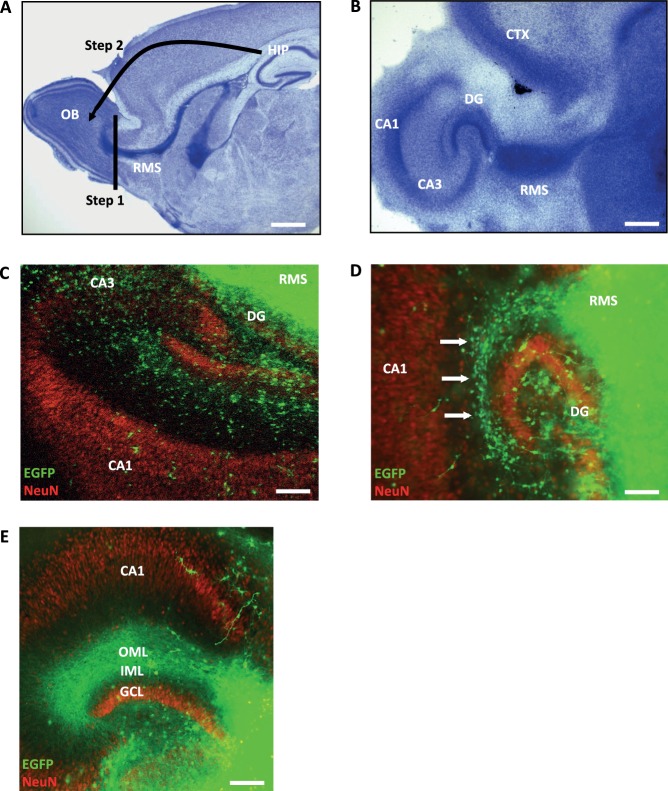
**Directing the RMS into the hippocampus in organotypic co-cultures.** (A) Experimental strategy to establish a new slice co-culture model. After explanting the forebrain, the OB is removed using a scalpel (Step 1). Immediately after that, a hippocampal (HIP) slice is prepared and connected to the RMS (Step 2). Scale bar, 500 μm. (B) Nissl stain showing a representative co-culture. Note the well-preserved anatomy of the hippocampus and the RMS, which remains a stream of migratory cells *in vitro*. Scale bar, 200 μm. (C) Chimeric co-culture consisting of wild type hippocampus and RMS from EGFP transgenic mice. Note that EGFP^+^ cells are widely distributed in the dentate gyrus (DG) and CA1–CA3 regions. Scale bar, 200 μm. (D) Higher magnification of a chimeric co-culture showing EGFP^+^ cells in the DG, hippocampal fissure, and CA1. Arrows highlight the stream of migratory cells in the hippocampal fissure. Scale bar, 100 μm. (E) Apart from RMS cells that migrate into the hippocampus, EGFP^+^ neurites also grow into the host tissue. Note that the outer molecular layer (OML) and inner molecular layer (IML) of the DG are densely packed with EGFP^+^ fibers. GCL, granule cell layer; scale bar, 200 μm;

Next, to probe the cellular plasticity of the RMS under organotypic tissue culture conditions, we asked if it would be feasible to direct migratory neuroblasts of the RMS into the hippocampus as a new destination. Hence, we prepared RMS-OB slices but removed the OB with a scalpel and immediately replaced it by a hippocampal slice (Fig. 2A). Remarkably, these RMS-hippocampal co-cultures could be maintained for several weeks, while the typical anatomy of both regions remained intact (Fig. 2B). To test if RMS cells could migrate into the hippocampus, we derived the RMS from transgenic mice ubiquitously expressing Enhanced Green Fluorescent Protein (EGFP) driven by the chicken β-actin promoter. Since hippocampal slices were prepared from wildtype mice (C57BL/6), any EGFP^+^ cell in the new host environment (hippocampus) should indicate the presence of RMS-derived donor cells. Indeed, these chimeric co-culture experiments showed that significant numbers of EGFP^+^ cells are capable of migrating into the hippocampus. Consistently, by DIV30 EGFP^+^ cells were widely distributed in the DG and CA1-CA3 areas (Fig. 2C,D). Based on our pilot experiments and the observation that RMS cells showed affinity to migrate into the hippocampus through the hippocampal fissure (Fig. 2D, arrows), we decided to place the RMS always next to the DG in all slice preparations. Hence, due to its proximity to the RMS, the DG was consistently the hippocampal subregion containing the largest number of EGFP^+^ cells (Fig. 2C,D, supplementary material Fig. S1C,D). In a different set of control experiments we addressed the question if cell migration is a general phenomenon that occurs in slice cultures. Hence, wild type hippocampus was either co-cultured with EGFP^+^ RMS (supplementary material Fig. S1A) or EGFP^+^ neocortical tissues that typically lack any RMS cells (supplementary material Fig. S1B). Because the hippocampus was virtually devoid of any EGFP^+^ neocortical cell at DIV30, we concluded that the RMS as a specific donor tissue is essential to introduce significant numbers of migratory EGFP^+^ cells into the hippocampus. Finally, we also observed that large numbers of EGFP^+^ neurites are capable of growing into the hippocampal host tissue. Fig. 2E demonstrates the formation of a dense band of EGFP^+^ neurites in the molecular layer of the DG.

**Fig. 3.**
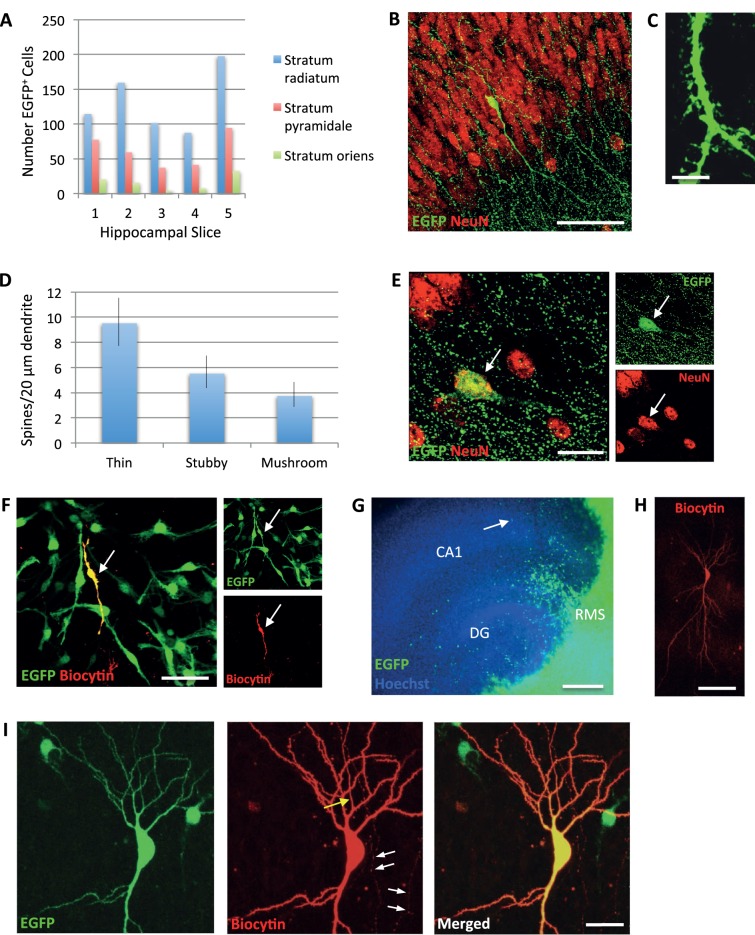
**Analysis of EGFP^+^ RMS cells in the hippocampal CA1 region.** (A) Distribution of EGFP^+^ cells in different CA1 layers. Cells were counted in five representative hippocampal slices after co-culture with the RMS for 30 days. (B) Overview of the CA1 region showing EGFP^+^ pyramidal-like neuron and widely distributed numerous EGFP^+^ bouton-like puncta. Scale bar, 50 μm. (C) Higher magnification of EGFP^+^ dendrite indicating the formation of numerous spine-like specializations. Scale bar, 5 μm. (D) Quantification of dendritic spines with thin, stubby, and mushroom morphologies (*n*=9 cells; mean±s.d.). (E) Confocal image showing NeuN expression by EGFP^+^ cell (arrow) in CA1 stratum pyramidale. Scale bar, 20 μm. (F) Biocytin-filled immature migratory EGFP+ cell (red pseudocolor) with bipolar morphology (arrow). Scale bar, 100μm. (G) Chimeric co-culture illustrating the distribution of RMS cells in the hippocampus. Note the stream of EGFP^+^ cells in the hippocampal fissure. Arrow indicates an EGFP^+^ cell in CA1 that was filled with biocytin (panel H). Scale bar, 200 μm. (H) Overview image of the biocytin-filled neuron highlighted in panel G (arrow). (I) Higher magnification shows the biocytin-filled cell presented in panels G and H. Yellow arrow points to the location of the axon initial segment and white arrows indicate axon collaterals traversing the neuropil. Scale bar, 100 μm.

Careful microscopic analysis of EGFP^+^ cells in the hippocampus at DIV30 showed that the reporter gene was variably expressed (Fig. 3; supplementary material Figs S2 and S3). Depending on the intensity and intracellular distribution of EGFP, cells with complex neuronal phenotypes and dendritic spines were detected suggesting neuronal differentiation of RMS cells in the hippocampus (Fig. 3B,C; supplementary material Figs S2C and S3B). Next, we profiled for the expression of neuronal markers using specific antibodies. We detected large numbers of cells with complex morphologies that co-expressed EGFP and different neuronal markers. In the DG, many of the EGFP^+^ cells expressed neurofilaments (NF-pan), calbindin (CB), calretinin (CR) or NeuN (supplementary material Figs S2 and S3A). Interestingly, some EGFP^+^ cells in the DG displaying neuronal morphologies were devoid of NeuN (supplementary material Fig. S3B). CR is transiently expressed by developing hippocampal granule cells, whereas CB is a marker for mature granule cells (Kempermann et al., 2004). However, both calcium-binding proteins are also expressed by subsets of hippocampal and olfactory interneurons (Freund and Buzsáki, 1996; Merkle et al., 2014) and cannot be taken as selective markers. We therefore performed immunolabeling for Prox1, a more specific marker for dentate granule cells (Knoth et al., 2010). Interestingly, the vast majority of EGFP^+^ cells were immunonegative for Prox1 (data not shown). Sporadically, only a few in the hippocampal fissure co-localized EGFP^+^ and Prox1 (supplementary material Fig. S3C). These experiments suggest that many RMS-derived cells differentiated into neurons expressing NF-pan, CB, CR, and NeuN, while only a few expressed the granule cell marker Prox1 under these experimental conditions.

Due to the anatomical orientation of slices during preparation, RMS cells had to migrate longer distances beyond the DG in order to reach the CA1 region (Fig. 2, supplementary material Fig. S1). Although we observed lower numbers of EGFP^+^ cells in CA1 as compared to the DG (Fig. 2C,D; supplementary material Fig. S1C,D), EGFP+ cells were consistently detected throughout CA1 (Fig. 3A). The neuronal identity of EGFP^+^ cells found in the CA1 stratum pyramidale (Fig. 3E, supplementary material Fig. S3D) and stratum radiatum (supplementary material Fig. S3E,F) was confirmed by NeuN expression. Some of these cells displayed long processes, complex dendritic arbors, and dendritic spines (Fig. 3B,C; supplementary material Fig. S3E,F). Furthermore, when using an antibody against GFP to amplify the signal, we found large amounts of EGFP^+^ bouton-like puncta throughout all hippocampal subregions (Fig. 3B,E) suggesting a wide distribution of axonal projections. It is likely that EGFP^+^ boutons arise from new cells in the hippocampus as well as from ingrowing neurites from the source tissue (Fig. 2E). To better characterize the morphology and differentiation of new cells in the CA1 region, we performed intracellular filling of EGFP^+^ cells in live slice cultures (Fig. 3F-I). Guided by fluorescence microscopy, EGFP^+^ cell bodies were identified and filled with biocytin in the whole-cell configuration. These experiments demonstrated that morphological analysis of biocytin-labeled cells allows distinguishing between undifferentiated and highly differentiated cells. For instance, cell filling of EGFP^+^ cells confirmed the presence of migratory RMS cells with bipolar morphology even after long-term culture (Fig. 3F). Remarkably, at the same time, in the pyramidal cell layer of CA1 we identified highly differentiated neuronal cells exhibiting basal and apical dendritic arbors (Fig. 3G-I). Cell fillings and confocal microscopy not only showed complex dendritic ramifications but also numerous dendritic spines decorating basal and apical dendrites. Furthermore, we were able to visualize the axons of biocytin-filled cells. Axonal profiles could be traced in the hippocampal slice over hundreds of microns (Fig. 3I, arrows). These results demonstrate that some RMS cells undergo long-distance migration and have the potential to differentiate into pyramidal-like neurons in the hippocampal CA1 region.

**Fig. 4.**
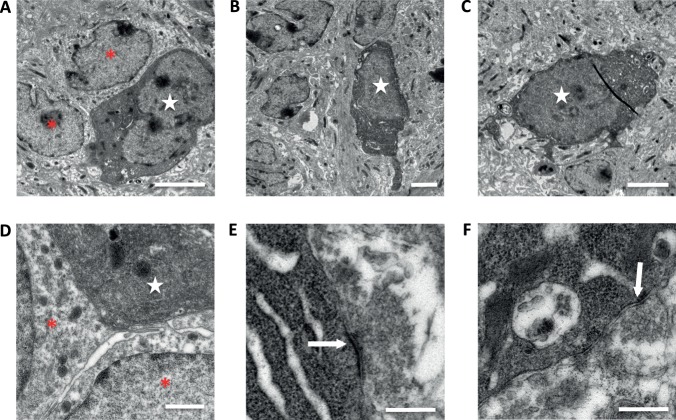
**Electron microscopic analysis of EGFP^+^ RMS cells in hippocampal CA1.** (A-D) Immuno-electron microscopic analysis identifies EGFP^+^ cells that display morphological features of pyramidal neurons (white stars). These examples illustrate that RMS-derived new EGFP^+^ pyramidal neurons are adjacent to unlabeled wild type pyramidal neurons of the host tissue (red asterisks). (E,F) Examples for newly formed synaptic connections (arrows). Presynaptic boutons contain numerous synaptic vesicles and contact EGFP^+^ postsynaptic structures. Scale bars, 0.5 μm.

Detection of EGFP^+^ bouton-like structures in the hippocampus suggested the formation of new synapses in the host tissue. Ultrastructural analysis can provide unequivocal evidence for cellular and synaptic integration. Hence, using an antibody against GFP and immunoperoxidase labeling, we processed chimeric slice co-cultures for electron microscopic analysis of. Analysis of the CA1 stratum pyramidale showed EGFP-labeled cells displaying ultrastructural characteristics of pyramidal neurons. Typical features of these cells were large round nuclei with homogenous karyoplasm, several dense bodies, and well-developed rough endoplasmic reticulum (Fig. 4A-D). Further careful analysis of EGFP^+^ structures suggested the formation of asymmetric synapses with prominent postsynaptic densities (Fig. 4E) as well as symmetric synapses without apparent postsynaptic specializations (Fig. 4F). Together, these findings support the notion that RMS-derived cells can differentiate into pyramidal-like neurons and integrate into the hippocampus.

Here, we established and analyzed a novel slice co-culture model by directly connecting the RMS to the hippocampus. We report that large numbers of RMS cells migrate, differentiate, and integrate into the hippocampus. Notably, RMS cells that have migrated in the CA1 region are capable of differentiating into pyramidal neurons with axonal projections. *In vivo*, the SVZ-RMS system is programmed to generate a variety of olfactory interneurons. These interneurons typically do not develop long axons but become integrated into dendro-dendritic local neuronal networks (Brill et al., 2009; Merkle et al., 2014). Interestingly, previous *in vivo* studies suggested that NSCs of the SVZ or parenchymal progenitors expressing the proteoglycan NG2^+^ may generate projection neurons and interneurons (Magavi et al., 2000; Arvidsson et al., 2002; Nakatomi et al., 2002; Dayer et al., 2005; Gordon et al., 2007; Ernst et al., 2014). Based on these studies, generation of brain region-specific neuronal cell types might be possible in the adult brain, particularly under disease conditions and after injury (e.g. laser ablation, stroke, neurodegeneration). However, the study of complex *in vivo* models is challenging and a standardized system to study cellular fate change under *in vivo*-like conditions has not been established.

Collectively, the slice co-culture model described here represents a valuable platform to streamline and systematically test the cellular plasticity of olfactory RMS cells under defined *in vitro* conditions. In the current study we analyzed RMS-hippocampal co-cultures after 4 weeks but longer culture periods should be feasible depending on experimental needs. Genetic fate-mapping experiments, the use of knockout and transgenic mice, and injury models should enable a broad range of innovative grafting experiments that maintain the organotypic microenvironment of both host and donor tissues. Elucidating the genetic determinants and environmental factors that control neurogenesis and neuronal subtype specification may leverage novel paradigms for cell replacement and brain repair.

## MATERIALS AND METHODS

### Slice culture preparation

Slice were derived from C57BL/6 wildtype (*n*=54) and JAX C57BL/6 TgN transgenic mice expressing EGFP under the control of the chicken β-actin promoter (*n*=87) at postnatal days 5–7 and prepared as described previously (Zhao et al., 2004). Briefly, brains were isolated and slices were sectioned using a McIlwain tissue chopper (350 μm) under sterile conditions (laminar air flow bench). Slices were cultured on semi-porous membranes (Millicell Cell Culture Inserts, CM30, Millipore) placed into 6-well plates (Corning) with 1 ml/well medium. In all experiments we cultured two slice co-cultures per Millicell membrane. The medium (25% heat-inactivated horse serum, 50% minimal essential medium, 25% Hank's balanced salt solution, 2 mM glutamine, pH 7.2) was changed every 2 days. All cultures were kept at regular incubator conditions (humidified 37°C, 5% CO_2_). All experiments were performed in agreement with the institutional guide for animal care.

### Double-labeling immunocytochemistry

Free-floating slice cultures (*n*=130) were fixed in 4% paraformaldehyde (PFA) for 30 min at room temperature (RT). After several washings with phosphate buffered saline (PBS), slices were treated with 3% bovine serum albumin and 0.3% Trition X-100 dissolved in PBS. Samples were then incubated in different combinations of primary antibodies (dissolved in PBS, 1% BSA, and 0.3% Triton) overnight at RT. After multiple washings with PBS, secondary antibodies (dissolved in PBS, 1% BSA, and 0.3% Triton) were applied for 6 h at RT. After nuclear staining with Hoechst 33258, slices were coverslipped in Mowiol/DABCO (Calbiochem) and analyzed for fluorescence signals. In control experiments primary or secondary antibodies were omitted.

### Antibodies

The following primary antibodies were used: mouse NeuN (1:500; Millipore); mouse Calbindin (1:1000; SWant); mouse Calretinin (1:250; BD Transduction Laboratories) rabbit Prox1 (1:500; Millipore); rabbit DCX (1:250; Santa Cruz); mouse NF-pan (1:3; Zymed); rabbit GFP (1:1000; Life Technologies). To detect primary antibodies, the following appropriate species-specific secondary antibodies were used: Goat anti-rabbit Alexa-488 (1:500; Life Technologies), Goat anti-mouse Alexa-568 (1:500; Life Technologies).

### BrdU experiment

To label proliferative cells, slices (*n*=12) were incubated for 24 h in BrdU (10 μM, Sigma). After fixation in 4% PFA, slices were incubated in 2 N HCl for 30 min at 37°C followed by washing in borate buffer (pH 8.5) for 15 min. Then a monoclonal rat-anti BrdU (1:1000; Harlan) and goat anti-rat Alexa-568 (1:500; Life Technologies) were used to visualize BrdU-labeled cells.

### Cell filling experiment

Whole-cell recordings were obtained using Axopatch 200B amplifier (Axon Instruments) with pipettes pulled from borosilicate glass tubing (2 mm outer diameter, 1 mm wall thickness). Pipettes were filled with an internal solution containing (in mM) 135 K gluconate, 20 KCl, 0.1 EGTA, 10 Hepes, 2 MgCl2, and 2 ATP (disodium salt) with 0.1% biocytin (Molecular Probes) added and had a resistance of 2–3 MΩ. EGFP-expressing neurons were visually identified using epifluorescent illumination and selected neurons were approached under visual control by infrared-differential interference contrast (IR-DIC) videomicroscopy. Neurons were filled for at least 15 min in the whole-cell configuration and an outside-out patch was obtained when withdrawing the electrode. Following overnight immersion fixation (4% PFA in 0.1 M phosphate buffer), biocytin-filled neurons were visualized using avidin conjugated Cy-3 (1:500; Life Technologies).

### Light and confocal microscopy

Slices were analyzed using a light microscope (Leica DM) and a confocal microscope (Leica TCS NT) equipped with standard filter sets.

### Electron microscopy

Electron microscopy was performed as described previously (Singec et al., 2002). Briefly, free-floating slices (*n*=24) were fixed in 4% PFA and 2.5% glutaraldehyde for 30 min. After several washings and blocking endogenous peroxidase with H_2_O_2_ (0.3%), free-floating slices were incubated in anti-GFP (1:1000) overnight. Triton X-100 was omitted in all steps in order to maintain ultrastructural integrity. Appropriate secondary antibody, avidin-biotinylated horseradish peroxidase, and 3,3-diaminobenzidine (Vectastain ABC Kit, Vector Laboratories) were used to visualize EGFP. Semi-thin sections were prepared to identify the CA1 region and ultrastructural analysis was performed using an electron microscope.
